# New-onset non-infectious pulmonary manifestations among patients with systemic lupus erythematosus in Sweden

**DOI:** 10.1186/s13075-018-1804-8

**Published:** 2019-02-06

**Authors:** Lindsy J. Forbess, Marios Rossides, Michael H. Weisman, Julia F. Simard

**Affiliations:** 1 Division of Rheumatology, Department of Medicine, Cedars-Sinai Medical Center, Los Angeles, CA USA; 2 Division of Clinical Epidemiology, Department of Medicine Solna, Karolinska Institutet, Stanford School of Medicine, Stockholm, Sweden; 3 Division of Epidemiology, Department of Health Research and Policy, Stanford School of Medicine, Stanford, CA USA; 4 Division of Immunology and Rheumatology, Department of Medicine, Stanford, CA USA

**Keywords:** Systemic lupus erythematosus, Pulmonary, Lung disease, Epidemiology

## Abstract

**Objective:**

The objective was to estimate the incidence of lung disease among patients with systemic lupus erythematosus (SLE).

**Methods:**

Using Swedish register data, we identified patients with SLE and pulmonary diagnoses from the National Patient Register through ICD codes. We matched patients with SLE with individuals from the general population. Patients with SLE with a history of pulmonary disease were excluded. Incidence rates (IR) and 95% confidence intervals (CI) were calculated overall and by type of pulmonary disease for incident (2003–2013) and prevalent SLE separately. Hazard ratios (HR) and 95% CI of the association between SLE and pulmonary disease were estimated using adjusted Cox regression models. Sensitivity analyses using a semi-automated approach to quantitative probabilistic bias analysis accounted for potential bias due to unmeasured confounding by smoking.

**Results:**

There were 3209 incident and 6908 prevalent cases of SLE identified. The IRs for pulmonary disease were similar in prevalent and incident SLE (∼14 cases per 1000 person-years). Patients with incident SLE had a nearly sixfold higher rate of pulmonary disease compared to the non-SLE population (HR 5.8 (95% CI 4.8–7.0)). Incident and prevalent SLE was associated with an increased rate of interstitial lung disease (HR 19.0 (95% CI 10.7–34.0) and 14.3 (95% CI 10.8–18.8), respectively). Bias due to unmeasured confounding by smoking was unlikely to explain our findings.

**Conclusion:**

Lung disease is relatively common in patients with SLE compared to the general population. Clinicians caring for patients with SLE should have heightened suspicion of lung disease, including interstitial lung disease, even early within the disease course or at the time of diagnosis of SLE.

**Electronic supplementary material:**

The online version of this article (10.1186/s13075-018-1804-8) contains supplementary material, which is available to authorized users.

## Significance and innovation

- This is the first population-based register study of lung disease in systemic lupus erythematosus (SLE) and shows that lung disease occurs at a much higher rate in patients with SLE compared to the general population
- Rates of interstitial lung disease were the same for incident and prevalent SLE, suggesting that patients with SLE in the early stages of their disease experience interstitial lung disease at similar rates to those with longer standing disease
- Clinicians caring for patients with SLE should have heightened suspicion of lung disease even early within the disease course

## Introduction

Systemic lupus erythematosus (SLE) is a heterogeneous autoimmune disease that has the potential to affect many organ systems. The lungs can be involved in up to two thirds of patients with SLE at some point in their disease course [1]. Prior studies have demonstrated a wide array of pulmonary manifestations, some of which are transient and not life threatening, such as pleurisy, to others that are more permanent and/or severe, such as interstitial lung disease (ILD) and pulmonary hemorrhage [1–3].

Much of what is known about lung disease in SLE comes from relatively small studies in selected clinical cohorts, such as the US multiethnic LUMINA cohort along with North American and European cohorts [1–3]. The true prevalence and incidence of lung disease in SLE may be underestimated in these studies because of patient selection, small sample sizes, and short follow-up periods. It is therefore important to identify pulmonary manifestations in a larger population-based cohort of individuals with SLE. Using such population-based data, less common phenotypes of SLE-related lung disease may be identified along with their onset relative to SLE diagnosis.

Therefore, we sought to investigate the prevalence and incidence of lung disease and whether lung manifestations occur early in SLE, using the Swedish Lupus Linkage (SLINK) cohort. It includes individuals with SLE and comparators from the general population who were identified through linkage of national registers. Using SLINK, we quantified and compared the history and incidence of pulmonary disease in individuals with SLE compared to those without SLE. This information will help us to understand the burden of lung disease among patients with SLE and if we can diagnose and potentially treat SLE lung manifestations early in the disease course to ultimately prevent pulmonary progression and damage. It will further help us to define a standard approach to detecting and treating lung disease in patients with SLE.

## Methods

### Study population and follow up

All individuals registered in Sweden with a personal identification number are eligible for the tax-funded universal health care. Inpatient (1964–2013) and outpatient non-primary care (2001–2013) data from the National Patient Register (NPR) were used to define SLE. Individuals of all ages with ≥ 2 visits listing an SLE-specific ICD code and ≥ 1 such visit registered with a relevant specialist (rheumatology, dermatology, nephology, internal medicine, or pediatrics) were defined as SLE cases. This case definition has been evaluated against nearly 1000 clinically confirmed well-characterized patients with SLE from four university-based rheumatology clinics and demonstrated face validity when compared to other population-based cohorts with validation studies where medical record reviews were performed [4, 5].

For the primary analysis of incident SLE, we used the above definition but only included cases if they first appeared in January 2003 or later (i.e. a minimum washout period of 2 years to exclude individuals with history of SLE). Everyone with SLE was matched on sex, birth year, and county of residence to up to five general population comparators (referred to as non-SLE) from the Total Population Register. A secondary analysis focused on prevalent SLE, which included all individuals satisfying the SLE case definition above. This secondary prevalent SLE population started contributing person-time from 1 January 2001 or the date that they fulfilled the SLE definition, whichever occurred later. Non-SLE comparators were required to be alive and living in Sweden at the time that their matched SLE case fulfilled the case definition (index date).

### Covariates

As described elsewhere, personal identification numbers were used to link data across multiple registers [6]. Briefly the study population was identified and linked to the NPR for all inpatient and outpatient non-primary care utilization and the Causes of Death Register (date and causes of death). Age and sex were identified from the Total Population Register. A proxy for age at SLE diagnosis was defined as age at first SLE coded visit in the NPR. Childhood-onset SLE was defined as age at start of SLE < 16 years and the remaining cases were classified as adult-onset SLE. Data were available from the Prescribed Drug Regsiter for individuals that entered the cohort starting in 2006, and medication use at inclusion (up to 90 days before the index date) was included in the following categories: glucocorticoids (prednisone, prednisolone, methylprednisolone, and betamethasone), hydroxychloroquine, and other disease-modifying anti-rheumatic drugs (DMARDs) including azathioprine, methotrexate, sulfasalazine, and mycophenolic acid.

### Pulmonary outcomes

Pulmonary diagnosis and date were identified using ICD codes from the NPR’s main and contributory discharge diagnoses in inpatient and outpatient visit data. Literature review was employed initially to guide the pulmonary outcomes typically seen among patients with SLE. Expert consensus among authors was used for the final pulmonary outcome ICD code list after the literature search did not reveal any additional information. Pulmonary diagnoses related to infection (i.e. bronchitis due to infectious etiologies) or cardiac causes (i.e. pulmonary edema) were excluded. In addition, codes that corresponded to “other diseases of the respiratory system not elsewhere specified” were excluded due to lack of specificity. Pulmonary outcomes were categorized as (a) interstitial lung disease (ILD), (b) acute respiratory distress syndrome and hemorrhage, (c) pleural disorders, (d) pulmonary hypertension, (e) pulmonary embolism, (f) diseases of the upper airway, and (g) pulmonary edema (not due to cardiac causes). ILD included non-specific interstitial pneumonia, organizing pneumonia, lymphoid interstitial pneumonia, usual interstitial pneumonia, pulmonary fibrosis, idiopathic pulmonary fibrosis, post-inflammatory pulmonary fibrosis, and acute interstitial pneumonitis. Pleural disorders included pleurisy and pleurisy with effusion. Diseases of the upper airway were bronchitis and bronchiectasis not due to infectious causes. The ICD codes used to identify these outcomes are provided in Additional file 1: Table S1.

Individuals contributed person-time from the index date until the first of the following: end of follow up (31 December 31 2013), pulmonary outcome of interest, or loss to follow up due to death or emigration. Individuals with a history of any of the pulmonary outcomes of interest were excluded from the study population. Our final study population included 3209 individuals with incident SLE between 2003 and 2013 and 17,658 non-SLE comparators free of history of pulmonary diagnoses at start of follow up. Expanding to prevalent SLE yielded 6908 individuals with SLE and 37,046 non-SLE individuals.

### Statistical analysis

All analyses were performed and reported separately for incident and prevalent SLE. Mean and median follow-up time was calculated for SLE cases and the non-SLE comparators. We estimated crude incidence rates (IRs) per 1000 person-years for pulmonary disease overall and for each type separately in our matched cohort. Age-adjusted and sex-adjusted Cox models were used to estimate hazard ratios (HRs) and corresponding 95% confidence intervals (CIs) comparing SLE to non-SLE.

We conducted probabilistic bias analyses [7] to examine the robustness of our analyses against unmeasured confounding by smoking - data that were not measured. Based on previous publications on SLE and smoking and nationwide health surveys conducted by the Public Health Agency of Sweden, we assumed a uniform distribution for the prevalence of smoking among individuals with SLE ranging from 22 to 32% and a uniform distribution for the prevalence of smoking among individuals without SLE ranging from 18 to 28% [8, 9]. We also assumed a relative risk of 1.6 between smoking and these pulmonary manifestations, based on the literature on idiopathic pulmonary fibrosis [10].

## Results

The SLE cases and general population comparators were predominantly adults and approximately 85% were female (Table 1). Among both incident and prevalent SLE cases starting follow up when prescription data were available (mid 2005 onwards) approximately 40% filled a prescription for glucocorticoids, 30% for hydroxychloroquine, and 12% for other DMARDs around the start of their follow up. Both incident SLE and their matched comparators contributed a median 5 years of follow-up time. We observed approximately 15 pulmonary complications per 1000 person-years among patients with incident SLE compared to fewer than 3 pulmonary complications per 1000 person-years among non-SLE comparators (Table 2). Individuals with recently diagnosed SLE had 12.2 additional cases of pulmonary disease per 1000 person-years compared to the general population. We found little difference by sex in any pulmonary diagnosis but were underpowered to look at sex differences for specific pulmonary diseases.

**Table 1:** Baseline characteristics of individuals with incident and prevalent systemic lupus erythematosus (SLE) and their matched general population comparators

	Incident SLE		Prevalent SLE	
	SLE	General population	SLE	General population
Subjects, *n*	3209	17,658	6908	37,046
Age at inclusion, mean (SD)	47 (19.1)	47 (18.6)	50 (18.1)	49 (17.7)
Female, %	85.7	84.7	86.8	85.9
Country of birth, %				
Nordic	84.8	89.6	89.1	91.1
Non-Nordic	15.2	10.3	10.8	8.9
Missing	< 1	< 1	< 1	< 1
Years of education, %				
≤ 9	27.8	24.0	30.4	26.8
10–12	40.0	41.1	41.0	42.1
≥ 13	27.1	30.1	24.9	27.3
Missing	5.1	4.7	3.7	3.9

**Table 2:** Incidence rates and hazard ratios for various lung diseases in individuals with incident systemic lupus erythematosus and general population comparators (2003–2013)

	Incident SLE (*n* = 3209)			General population (*n* = 17,658)			
	Cases, *n* (%)	Person-years	Incidence rate per 1000 person-years (95% CI)	Cases, *n* (%)	Person-years	Incidence rate per 1000 person-years (95% CI)	Adjusted hazard ratio (95% CI)^a^
Any pulmonary disease	227 (7.1)	15,208	14.9 (13.1–17.0)	246 (1.4)	90,374	2.7 (2.4–3.1)	5.8 (4.8–7.0)
By specific pulmonary disease							
Interstitial lung disease	48 (1.5)	15,535	3.1 (2.3–4.1)	15 (0.1)	90,738	0.2 (0.1–0.3)	19.0 (10.7–34.0)
ARDS and hemorrhage	16 (0.5)	15,672	1.0 (0.6–1.7)	18 (0.1)	90,704	0.2 (0.1–0.3)	5.3 (2.7–10.5)
Pleural disorders	91 (2.8)	15,400	5.9 (4.8–7.3)	93 (0.5)	90,576	1.0 (0.8–1.3)	5.9 (4.4–7.9)
Pulmonary hypertension	18 (0.6)	15,675	1.2 (0.7–1.8)	18 (0.1)	90,737	0.2 (0.1–0.3)	6.1 (3.2–11.8)
Pulmonary embolism	49 (1.5)	15,568	3.2 (2.4–4.2)	78 (0.4)	90,595	0.9 (0.7–1.1)	3.8 (2.6–5.4)
Diseases of the upper airway	15 (0.5)	15,655	1.0 (0.6–1.6)	29 (0.2)	90,685	0.3 (0.2–0.5)	3.1 (1.7–5.8)
Pulmonary edema	8 (0.3)	15,703	0.5 (0.3–1.0)	15 (0.1)	90,752	0.2 (0.1–0.3)	3.3 (1.4–7.8)

*SLE* systemic lupus erythematosus, *CI* confidence interval

^a^Model adjusted for age (continuous) and sex

In incident SLE, overall pulmonary disease occurred at a nearly sixfold higher rate compared to the general population (HR 5.8 (95% CI 4.8–7.0)). We found little difference by sex in the adjusted models (data not shown). The relative risk of ILD and pleural disorders compared to the general population was even higher among those with incident SLE than among those with prevalent SLE. There was a 19-fold increased rate of ILD, although with wide confidence intervals (95% CI 10.7–34.0), and an almost 6-fold increased rate of pleural disorders (95% CI 4.4–7.9) among incident SLE compared with the general population. Rates of other specific pulmonary diseases did not change much among incident SLE compared with those with prevalent SLE but were much higher compared to the general population (Tables 2 and 3).

**Table 3:** Incidence rates and hazard ratios for various lung diseases in individuals with prevalent systemic lupus erythematosus and general population comparators (2001–2013)

	Prevalent SLE (*n* = 6908)			General population (*n* = 37,046)			
	Cases, *n* (%)	Person-years	Incidence rate per 1000 person-years (95% CI)	Cases, n (%)	Person-years	Incidence rate per 1000 person-years (95% CI)	Adjusted hazard ratio (95% CI)^a^
Any pulmonary disease	700 (10.1)	51,048	13.7 (12.7–14.8)	872 (2.4)	310,538	2.8 (2.6–3.0)	5.4 (4.9–6.0)
By specific pulmonary disease							
Interstitial lung disease	170 (2.5)	52,355	3.3 (2.8–3.8)	73 (0.2)	312,193	0.2 (0.2–0.3)	14.3 (10.8–18.8)
ARDS and hemorrhage	51 (0.7)	52,954	1.0 (0.7–1.3)	54 (0.2)	312,245	0.2 (0.1–0.2)	5.8 (3.9–8.5)
Pleural disorders	237 (3.4)	52,083	4.6 (4.0–5.2)	317 (0.9)	311,498	1.0 (0.9–1.1)	4.6 (3.9–5.5)
Pulmonary hypertension	63 (0.9)	52,970	1.2 (0.9–1.5)	61 (0.2)	312,310	0.2 (0.2–0.3)	6.8 (4.7–9.6)
Pulmonary embolism	174 (2.5)	52,544	3.3 (2.9–3.8)	293 (0.8)	311,543	0.9 (0.8–1.1)	3.8 (3.1–4.6)
Diseases of the upper airway	62 (0.9)	52,841	1.2 (0.9–1.5)	92 (0.3)	312,066	0.3 (0.2–0.4)	4.2 (3.0–5.8)
Pulmonary edema	34 (0.5)	53,094	0.6 (0.5–0.9)	53 (0.1)	312,380	0.2 (0.1–0.2)	4.3 (2.8–6.7)

*SLE* systemic lupus erythematosus, *CI* confidence interval, *ARDS* acute respiratory distress syndrome

^a^Model adjusted for age (continuous) and sex

Among those with prevalent SLE, approximately 10% had at least one pulmonary manifestation during follow up compared with 2.4% in the general population (Table 3). The highest incidence rate was observed for pleural disorders (4.6 (95% CI 4.0–5.2)) followed by pulmonary embolism and ILD (3.3 (95% CI 2.9–3.8) for both). Having SLE was associated with a 5.4-fold increased risk of any pulmonary disease during follow up (95% CI 4.9–6.0) in adjusted models comparing to the general population. When considering each pulmonary disease group separately, the highest relative risk was found for ILD (HR 14.3 (95% CI 10.8–18.8)) and the lowest for pulmonary embolism (HR 3.8 (95% CI 3.1–4.6); Table 2). Due to limited power (< 5 cases) we were unable to investigate pediatric risks separately.

Bias analyses suggested that the HR from the main analyses was robust to unmeasured confounding due to smoking even under extreme confounding conditions. This was the case for both incident SLE (HR 5.5 (95% simulation interval 4.5–6.6)) and prevalent SLE (HR 5.1 (95% simulation interval 4.6–5.6)). These results did not change appreciatively when assuming a substantially higher relative risk of 5.4 between smoking and these pulmonary manifestations.

## Discussion

In this large population-based investigation, we have presented results on the incidence of various pulmonary disease manifestations in patients with incident and prevalent SLE. Our analyses showed that there is a much higher rate of overall pulmonary disease in both prevalent and incident cases of SLE compared to the general population. After adjusting for age and sex, there was an almost sixfold increased rate of pulmonary disease among those with incident SLE and a fivefold increased rate of pulmonary disease among those with prevalent SLE compared to the general population. The largest relative risk was for ILD, which appears to be partly driven by the observed low incidence of ILD in the general population.

The incidence rates were similar for all the individual pulmonary outcomes for incident and prevalent SLE. It is particularly intriguing that the rates of ILD for incident SLE were essentially the same as for prevalent SLE (just over three ILD cases per 1000 person-years of follow up). This does not imply that survival is poor but rather suggests that patients in the early stages of SLE experience ILD at a similar rate to patients with prevalent SLE. Data from the literature confirm that pulmonary damage is relatively common in SLE. In the multiethnic LUMINA cohort of over 600 patients with SLE, 5-year and 10-year cumulative risks of pulmonary damage were 7.6% and 11.6%, respectively [3].

Our study has some limitations. We cannot exclude that there may be some misclassification of SLE and pulmonary outcomes. We used a robust definition of SLE requiring multiple visits with a SLE code along with a diagnosis from a specialist. We anticipate that misclassification is minimal [4] and, if present, would most likely dilute the associations we observed towards the null. Pulmonary outcomes (i.e. ILD, pulmonary embolism) have previously been defined similarly using discharge diagnoses [11, 12] along with validation studies showing that the majority of cases met objective diagnostic criteria [13, 14]. Some pulmonary manifestations, including subtypes of ILD, are more difficult to validate and we cannot exclude the possibility of some misclassification.

We did not have data on SLE phenotypes (such as neuropsychiatric lupus or lupus nephritis), disease activity, or antiphospholipid syndrome or antibody status. By incorporating outpatient and inpatient admissions in our SLE definition, a range of phenotypes and disease severities were likely captured. In addition, our study was conducted using a relatively homogenous population in Sweden and therefore may not be generalizable to non-Caucasian populations with potentially different patterns of SLE disease manifestations and severity. Despite the relatively large study population, we had too few events to study outcomes in pediatric SLE separately. This would be interesting for a future study given a recent meta-analysis highlighting the fact that pulmonary manifestations of SLE were more common in patients with SLE who presented after the age of 50 compared to their younger peers, particularly ILD and serositis [15]. Last, we also did not have data on the smoking habits of our population. However, we conducted sensitivity analysis to assess the scope of unmeasured confounding by smoking and showed that our estimates from the main analyses were robust even under extreme bias assumptions.

Using Swedish population-based registers was a major strength of this investigation. We were able to obtain a representative sample of patients with SLE who together with their matched comparators from the general population could be followed with minimal losses. Our results are readily generalizable to other adult populations receiving similar standards of care to the Swedish population. Furthermore, the use of general population comparators is a strength, as it neither requires that individuals be sick with another disease, nor selects “healthy” individuals, both of which would distort the comparison. Last, we considered both prevalent and incident SLE to evaluate pulmonary disease complications in patients with established and newly identified disease, respectively.

## Conclusion

This was the first population-based study of its kind addressing lung disease in SLE. We demonstrated that the rate of lung disease is much higher in SLE compared to the general population. Clinicians caring for patients with SLE should have heightened suspicion of lung disease, including ILD, even early within the disease course. We cannot say with certainty from our analyses that these findings of lung disease are clinically significant or important. However, additional investigations could define an important natural history of these findings and later help facilitate early intervention and prevention of pulmonary damage if it occurs.

## Additional file

Additional file 1: **Table S1.** Swedish ICD-10/9/8 codes used to retrieve the data from the Swedish National Patient Register. (DOCX 14 kb)

## Abbreviations

ILD: Interstitial lung disease
IR: Incidence rate
NPR: National Patient Register
SLE: Systemic lupus erythematosus
SLINK: Swedish Lupus Linkage

## References

[1] Allen D, Fischer A, Bshouty Z, Robinson DB, Peschken CA, Hitchon C (2012). Evaluating SLE patients for lung involvement. Lupus.

[2] Cervera R, Khamashta MA, Font J, Sebastiani GD, Gil A, Lavilla P (2003). Morbidity and mortality in systemic lupus erythematosus during a 10-year period: a comparison of early and late manifestations in a cohort of 1,000 patients. Medicine (Baltimore).

[3] Bertoli AM, Vila LM, Apte M, Fessler BJ, Bastian HM, Reveille JD (2007). Systemic lupus erythematosus in a multiethnic US Cohort LUMINA XLVIII: factors predictive of pulmonary damage. Lupus.

[4] Arkema EV, Jonsen A, Ronnblom L, Svenungsson E, Sjowall C, Simard JF (2016). Case definitions in Swedish register data to identify systemic lupus erythematosus. BMJ Open.

[5] Simard JF, Sjowall C, Ronnblom L, Jonsen A, Svenungsson E (2014). Systemic lupus erythematosus prevalence in Sweden in 2010: what do national registers say?. Arthritis Care Res.

[6] Arkema EV, Simard JF (2015). Cohort profile: systemic lupus erythematosus in Sweden: the Swedish Lupus Linkage (SLINK) cohort. BMJ Open.

[7] Last TL, Fink AK (2003). Semi-automated sensitivity analysis to assess systematic errors in observational data. Epimediology.

[8] Costenbader KH, Kim DJ, Peerzada J, Lockman S, Nobles-Knight D, Petri M (2004). Cigarette smoking and the risk of systemic lupus erythematosus: a meta-analysis. A&R.

[9] Folkhälsomyndigheten. (The Public Health Agency of Sweden). Information on smoking (in Swedish): https://www.folkhalsomyndigheten.se/livsvillkor-levnadsvanor/alkohol-narkotika-dopning-tobak-och-spel-andts/tobak/. Accessed June 2018.

[10] U.S. Department of Health and Human Services. The health consequences of smoking: 50 years of progress. A Report of the Surgeon General. Atlanta, GA: U.S. Department of Health and Human Services, Centers for Disease Control and Prevention, National Center for Chronic Disease Prevention and Health Promotion, Office on Smoking and Health, 2014. Printed with corrections, January 2014.

[11] Sode BF, Dahl M, Nielsen SF, Nordestgaard BG (2010). Venous thromboembolism and risk of idiopathic interstitial pneumonia. Am J Respir Crit Care Med.

[12] Raghu G, Weycker D, Edelsberg J, Bradford WZ, Oster G (2006). Incidence and prevalence of idiopathic pulmonary fibrosis. Am J Respir Crit Care Med.

[13] Juul K, Tybjaerg-Hansen A, Schnohr P, Nordestgaard BG (2004). Factor V Leiden and the risk for venous thromboembolism in the adult Danish population. Ann Intern Med.

[14] Navaratnam V, Fogarty AW, Glendening R, McKeever T, Hubbard RB (2013). Hospital admission trends in England from 1998 to 2010. Chest.

[15] Medlin JL, Hansen KE, McCoy SS, Bartels CM. Pulmonary manifestations in late versus early systemic lupus erythematosus: a systematic review and meta analysis. Semin Arthritis Rheum. 10.1016/j.semarthrit.2018.01.010. 10.1016/j.semarthrit.2018.01.010 PMC6067995 29550111

