# Supplementary material for: New-onset non-infectious pulmonary manifestations among patients with systemic lupus erythematosus in Sweden
Source: Arthritis Res Ther. 2019 Feb 6;21:48. doi: 10.1186/s13075-018-1804-8 (PMC6366020; doi:10.1186/s13075-018-1804-8)
Supplement: Supplementary file 1 — Table S1. Swedish ICD-10/9/8 codes used to retrieve the data from the Swedish National Patient Register. (DOCX 14 kb) [file 13075_2018_1804_MOESM1_ESM.docx]

**Table S1** Swedish ICD-10/9/8 codes used to retrieve the data from the Swedish National Patient Register.

|  | **Swedish ICD coding system** | | |
| --- | --- | --- | --- |
|  | **version 10** | **version 9** | **version 8** |
| Interstitial lung disease (ILD)^a^ | J84, J99 | 516, 517 | 485,02; 486,01‒486,09; 517 |
| ARDS and hemorrhage | J80, R04.2, R04.8, R04.9 | 518F, 786D | 776,10; 783,10 |
| Pleural disorders^b^ | J90, J91, J94.8, J94.9, R09.1 | 511A, 511W‒511X | 511,01; 511,09;  511,10‒511,20 |
| Pulmonary hypertension | I27.0, I27.2‒ I27.9 | 416A, 416B, 416X | 426 |
| Pulmonary embolism | I26 | 415, 416W | 450 |
| Diseases of the upper airway^c^ | J41, J42, J47 | 491, 494 | 490; 491; 518,99 |
| Pulmonary edema | J81 | 514 | 514,99 |

^a^ILD = non-specific interstitial pneumonia, organizing pneumonia, lymphoid interstitial pneumonia, usual interstitial pneumonia, pulmonary fibrosis, idiopathic pulmonary fibrosis, post-inflammatory pulmonary fibrosis, and acute interstitial pneumonitis

^b^Pleural disorders = pleurisy, pleurisy with effusion

^c^Diseases of the upper airway = bronchitis and bronchiectasis not due to infectious causes
